# Comparison of the Efficacy and Safety of PD‐1/PD‐L1 Inhibitors in the Treatment of Small Cell Lung Cancer

**DOI:** 10.1002/cnr2.70081

**Published:** 2025-01-13

**Authors:** Qichen Zhang, Xiaojuan Han, Jiayi Liu, Hui Qiao

**Affiliations:** ^1^ The First Clinical Medical College of Lanzhou University Lanzhou China; ^2^ Department of Oncology The First Hospital of Lanzhou University Lanzhou China; ^3^ Gansu Province Clinical Research Center for Thoracic Tumor Lanzhou China

**Keywords:** adverse reactions, efficacy evaluation, PD‐L1/PD‐1, small cell lung cancer (SCLC)

## Abstract

**Objective:**

This study aims to evaluate the efficacy and safety of PD‐1/PD‐L1 inhibitors in treating small‐cell lung cancer (SCLC) and determine the role of PD‐1 monoclonal antibodies in improving patient outcomes.

**Methods:**

A retrospective analysis was performed on 37 SCLC patients who received PD‐1 or PD‐L1 inhibitors along with chemotherapy at the First Hospital of Lanzhou University between June 2018 and June 2023. Treatment effectiveness was measured by overall response rate (ORR), disease control rate (DCR), overall survival (OS), and progression‐free survival (PFS), utilizing chi‐square and T‐tests, along with Kaplan–Meier and log‐rank analyses.

**Results:**

In the PD‐L1 group, 16 patients achieved partial or complete response, versus 12 in the PD‐1 group, though the difference in the ORR was not statistically significant (50.0% vs. 36.8%, *p* = 0.308). Median survival times were 21.0 months for PD‐L1 and 17.0 months for PD‐1, with no statistically meaningful difference (*p* = 0.180). Adverse effects were comparable between the groups in terms of thyroid function (*p* = 0.898), but bone marrow suppression and gastrointestinal reactions were significantly less severe in the PD‐L1 group (*p* = 0.047 and *p* = 0.002).

**Conclusion:**

Immunotherapy combined with chemotherapy offers significant benefits for advanced SCLC patients, irrespective of the type of inhibitor used. Despite the higher incidence of adverse reactions with PD‐1 inhibitors, they remain a viable option, particularly when PD‐L1 inhibitors are not available, due to their manageable safety profile and effective response.

## 1 Introduction

Lung cancer is the leading cause of cancer incidence and mortality worldwide. According to the American Cancer Society (ACS), lung cancer comprised 14% of all cancer cases globally in 2020 and was responsible for 18% of cancer‐related deaths during the same year. This malignancy has a notably higher fatality rate compared to other cancer types [1].

Lung cancer is primarily classified into two histological types: non‐small‐cell lung cancer (NSCLC), which accounts for about 85% of cases, and small‐cell lung cancer (SCLC), which makes up the remaining 15% [2, 3]. Despite representing a smaller fraction of lung cancer cases, SCLC is characterized by its neuroendocrine nature, exhibiting aggressive behavior such as rapid growth rates, high invasiveness, and a tendency for early metastasis post‐diagnosis. These factors contribute to a higher mortality rate in SCLC compared to NSCLC [4]. Despite representing a smaller fraction of lung cancer cases, SCLC is characterized by its neuroendocrine nature, exhibiting aggressive behavior such as rapid growth rates, high invasiveness, and a tendency for early metastasis post‐diagnosis. These factors contribute to a higher mortality rate in SCLC compared to NSCLC [5]. Data show that patients with LS SCLC typically have a median overall survival (OS) of 15 to 20 months, while those in the ES SCLC group often see a much shorter median OS of 8 to 10 months [6]. It is common for around two‐thirds of SCLC patients to be diagnosed at the extensive stage during their initial evaluation, highlighting the rapid progression of the disease [7]. Traditionally, before the introduction of immunotherapy, the established first‐line treatment for ES SCLC involved the EP regimen—a combination of etoposide with a platinum‐based compound (cisplatin or carboplatin). This regimen has been a standard for more than three decades. Although the objective response rate (ORR) can reach up to 70%, the median PFS remains around 5.5 months, with the median OS not exceeding 10 months [8].

Recent research has highlighted the significance of immunotherapy and the mechanisms of immune evasion as pivotal areas in oncological research [9]. The investigation into agents targeting the PD‐1/PD‐L1 pathway has been of particular interest, highlighting its pivotal role in the realm of immune checkpoint modulation. This pathway functions predominantly by suppressing T lymphocyte activation and attenuating the host's immune response and enabling tumor cells to evade immune recognition and destruction, thus facilitating tumor growth and progression [10]. In 2022, the Chinese Society of Clinical Oncology (CSCO) updated their guidelines to include specific diagnostic and therapeutic recommendations for SCLC, with a particular focus on ES SCLC. These guidelines underscore the importance of tailored treatment approaches to address the aggressive nature of ES SCLC, reflecting advancements in understanding and managing this challenging condition. The guidelines advocate three first‐line treatment regimens: (1) carboplatin + etoposide + atezolizumab, (2) carboplatin + etoposide + durvalumab, and (3) cisplatin + etoposide + durvalumab [11]. In addition, the domestic humanized PD‐L1 inhibitor SHR‐1316 (Adebrelimab) also passed the marketing authorization application of the National Medical Products Administration (NMPA) of China in March 2023 [12, 13]. Compared with the remarkable effect of PD‐L1 inhibitors in SCLC treatment, the progress of PD‐1 inhibitors in SCLC treatment is slower. The PD‐1 inhibitor, Serplulimab, developed by Chinese researchers, received approval for the treatment of ES SCLC in April 2022 [14]. The efficacy of other PD‐1 inhibitors for ES SCLC is mostly still in the research stage. In terms of price, most PD‐L1 inhibitors are imported drugs, and their treatment costs are much higher than those of PD‐1 inhibitors, which have not been included in China's medical insurance yet. However, numerous domestic PD‐1 inhibitors are now covered by medical insurance, making them more accessible and affordable. Affected by economic factors, most patients tend to choose PD‐1 drugs [15]. This research aims to assess the effectiveness and safety of integrating PD‐1/PD‐L1 inhibitors with chemotherapy for treating patients with small‐cell lung cancer (SCLC), and investigates the potential clinical benefits of these inhibitors within the SCLC treatment framework.

Recent advances have demonstrated that immune checkpoint inhibitors, particularly those targeting PD‐L1, are effective in managing SCLC. Consequently, the combination of immunotherapy and chemotherapy has emerged as a potent therapeutic strategy for this disease [16].

## 2 Materials and Methods

### 2.1 Patients

A retrospective study was performed using clinical records from 37 small‐cell lung cancer (SCLC) patients treated with PD‐1 and PD‐L1 inhibitors at the First Hospital of Lanzhou University between June 2018 and June 2023. Data on baseline characteristics such as gender, age, smoking history, clinical stage, and Karnofsky Performance Status (KPS) score were meticulously gathered and analyzed for both patient groups.

### 2.2 Inclusion Criteria

(1) A confirmed diagnosis of SCLC through histology or cytology; (2) At least one measurable lung lesion was required; (3) Treatment with PD‐1/PD‐L1 inhibitors for 1 cycle or more; (4) Patients who have not received relevant prior immunotherapy.

### 2.3 Exclusion Criteria

(1) Patients with poor treatment compliance; (2) Patients with significant diseases affecting other organs; (3) Patients with contraindications to the drugs used in this study; (4) Patients unable to tolerate the treatment.

### 2.4 Research Indicators

#### 2.4.1 Response Evaluation Criteria in Solid Tumors(RECIST)

A comparison of pre‐ and post‐treatment imaging assessments was conducted based on the solid tumor evaluation criteria issued by the World Health Organization (WHO) [17]. The categorizations are as follows: (1) CR (complete response): Disappearance of lesions, normal tumor marker levels, and a duration exceeding four weeks. (2) Partial response (PR): Characterized by a decrease of at least 30% in the total size of the target lesions, maintained for a minimum duration of four weeks. (3) Stable disease (SD): Defined as a minor decrease in the total size of the target lesions that do not qualify as partial response (PR), or a slight increase that does not reach the threshold for progressive disease (PD). (4) Progressive disease (PD): Characterized by an expansion in the total size of the target lesions by at least 20% or the emergence of new lesions.

Key evaluation metrics utilized include:

1. **Objective Response Rate (ORR)**: This is derived by dividing the sum of patients achieving complete response (CR) and partial response (PR) by the total number of patients assessed. The formula used is: ORR = (CR + PR) / Total Evaluable Patients × 100%.
2. **Disease Control Rate (DCR)**: This encompasses all cases exhibiting CR, PR, or stable disease (SD). It is calculated as: DCR = (CR + PR + SD) / Total Evaluable Patients × 100%.
3. **Progression‐Free Survival (PFS)**: Measured from the time of initial study enrollment until the occurrence of tumor progression or death, whichever comes first.
4. **Overall Survival (OS)**: Defined as the interval between the commencement of treatment randomization and death from any cause.”

#### 2.4.2 Adverse Reactions

All patient discomforts during and post‐treatment were observed and recorded, including gastrointestinal, hepatic, respiratory, and bone marrow reactions, as well as thyroid dysfunction and more.

### 2.5 Statistical Evaluations Performed Using SPSS Software, Version 25.0

Continuous variables that followed a normal distribution were expressed as means and standard deviations (mean ± SD), with group comparisons conducted via independent t‐tests. Categorical variables were presented as frequencies and percentages and analyzed using chi‐square tests. Survival metrics, including OS and PFS, were calculated through the Kaplan–Meier approach, and discrepancies in survival rates across different groups were examined using log‐rank tests. Statistical significance was established at a p‐value below 0.05.

## 3 Results

### 3.1 Baseline Characteristics

Of the 37 SCLC patients, 19 were treated with PD‐1 inhibitors combined with chemotherapy, comprising 15 males and four females, averaging 56.05 ± 7.18 years in age. The majority (*n* = 11, 57.8%) were at WHO clinical stage IV, with 7 at Stage III and 1 at Stage I. Their KPS scores were predominantly 80, except for one case at 70. The remaining 18 patients received PD‐L1 inhibitors combined with chemotherapy, including 12 males and six females, with an average age of 57.94 ± 7.36 years. Most were at Stage IV (*n* = 13, 72.3%), 3 at Stage III, 1 at Stage II, and 1 at Stage I. Their KPS distribution was 1 at 70, 16 at 80, and 1 at 90. Comparative evaluations indicated that there were no statistically significant discrepancies in the baseline characteristics across the groups (*p* > 0.05). Table 1 summarizes the baseline characteristics.

**TABLE 1:** Initial Patient Demographics and Clinical Features in the Small Cell Lung Cancer Cohort.

Characteristics		PD‐1(*n* = 19)	PD‐L1(*n* = 18)	χ^2^	P
Age (years)				2.058	0.560
	41 ~ 50	5 (26.3%)	3 (16.7%)		
	51 ~ 60	9 (47.4%)	7 (38.9%)		
	61 ~ 70	5 (26.3%)	7 (38.9%)		
	≥ 71	0	1 (5.5%)		
		56.05 ± 7.18	57.94 ± 7.36		
KPS				1.091	0.579
	70	1 (5.3%)	1 (5.5%)		
	80	18 (94.7%)	16 (89.0%)		
	90	0	1 (5.5%)		
		79.47 ± 2.29	80.00 ± 3.43		
Gender				0.638	0.476
	Male	15 (78.9%)	12 (66.7%)		
	Female	4 (21.1%)	6 (33.3%)		
Clinical staging				2.742	0.433
	I	1 (5.3%)	1 (5.5%)		
	II	0	1 (5.5%)		
	III	7 (36.9%)	3 (16.7%)		
	IV	11 (57.8%)	13 (72.3%)		

The baseline characteristics: no significant differences in baseline characteristics between the two groups.

### 3.2 Comparison of Adverse Reactions

#### 3.2.1 Bone Marrow Suppression Level

In the PD‐1 cohort, bone marrow suppression was observed in all members. Specifically, in the PD‐1 cohort, there were three instances of grade 1, 7 of grade 2, 6 of grade 3, and 3 of grade 4 bone marrow suppression. In contrast, within the PD‐L1 cohort, four individuals showed no signs of bone marrow suppression, while 6 experienced grade I, 6 grade II, 1 grade III, and 1 grade IV suppression. A significant disparity was identified between the two groups (χ^2^ = 9.096, *p* = 0.047 < 0.05)(Table 2).

**TABLE 2:** Overview of Select Adverse Events Related to Treatment in a Cohort of 37 Patients Evaluated for Toxicity.

Adverse event		PD‐1(*n* = 19)	PD‐L1(*n* = 18)	χ^2^	P
Myelosuppression				9.096	0.047
	0	0	4 (22.2%)		
	1	3 (15.8%)	6 (33.3%)		
	2	7 (36.8%)	6 (33.3%)		
	3	6 (31.6%)	1 (5.6%))		
	4	3 (15.8%)	1 (5.6%)		
Gastrointestinal reaction				14.377	0.002
	0	0	7 (38.9%)		
	1	8 (42.1%)	9 (50.0%)		
	2	5 (26.3%)	2 (11.1%)		
	3	5 (26.3%)	0		
	4	1 (5.3%)	0		
Thyroid dysfunction				0.016	0.898
	Yes	12 (63.2%)	11 (61.1%)		
	No	7 (36.8%)	7 (38.9%)		

*Note:* Differential analysis of PD‐1/PD‐L1 adverse reactions showed that patients exhibited significant differences in myelosuppression and gastrointestinal reactions, with patients in the PD‐1 group experiencing significantly greater severity. However, the two groups did not show significant differences in terms of thyroid dysfunction.

#### 3.2.2 Gastrointestinal Reaction Severity

Typical gastrointestinal manifestations encompassed symptoms such as nausea, vomiting, and diarrhea. In the PD‐1 cohort, all manifested varying intensities of these symptoms: Within the PD‐1 cohort, adverse reactions were classified as follows: three individuals experienced grade 1, seven had grade 2, six underwent grade 3, and three faced grade 4 reactions. Conversely, in the PD‐L1 cohort, four participants exhibited no gastrointestinal symptoms, six showed grade 1, six had grade 2, one demonstrated grade 3, and one experienced grade 4 reactions. A significant variance in the severity of gastrointestinal reactions between the cohorts was evident (χ^2^ = 14.377, *p* = 0.002). (χ^2^ = 14.377, *p* = 0.002 < 0.05) (Table 2).

#### 3.2.3 Thyroid Dysfunction

The number of patients with thyroid dysfunction in the PD‐1 group and the PD‐L1 group was 12 and 11, respectively (63.2% vs. 61.2%). There was no statistically significant difference between the two groups of patients (χ^2^ = 0.016, *p* = 0.898 > 0.05) (Table 2).

### 3.3 Comparing Treatment Efficacy in Both Groups

#### 3.3.1 Efficacy Evaluation

Following treatment, the PD‐L1 group saw nine patients achieving either PR or CR, whereas the PD‐1 group had seven patients reaching these response levels. The ORR stood at 50.0% for the PD‐L1 group and 36.8% for the PD‐1 group. Nonetheless, the variation in response rates between the groups did not reach statistical significance.(χ^2^ = 3.646, *p* = 0.308 > 0.05)(Table 3).

**TABLE 3:** Confirmed Efficacy Results in the Total Populat.

Group	Efficacy No. (%)				ORR*(%)	DCR^a^(%)
	CR	PR	SD	PD		
PD‐1(*n* = 19)	2 (10.5)	5 (26.3)	5 (26.3)	7 (36.8)	36.8	63.2
PD‐L1(*n* = 18)	1 (5.6)	8 (44.4)	1 (5.6)	8 (44.4)	50.0	55.6
χ^2^					0.652	0.222
P					0.515	0.743

*Note:* Patient survival information is described in this table.

Abbreviations: CR: Complete Response; OS: Overall Survival; PFS: Progression‐Free Survival; PR: Partial Response; DCR: Disease Control Rate; ORR: Objective Response Rate”*ORR is CR + PR.

^a^ DCR is CR + PR + SD.

#### 3.3.2 Survival Time

**FIGURE 1:**
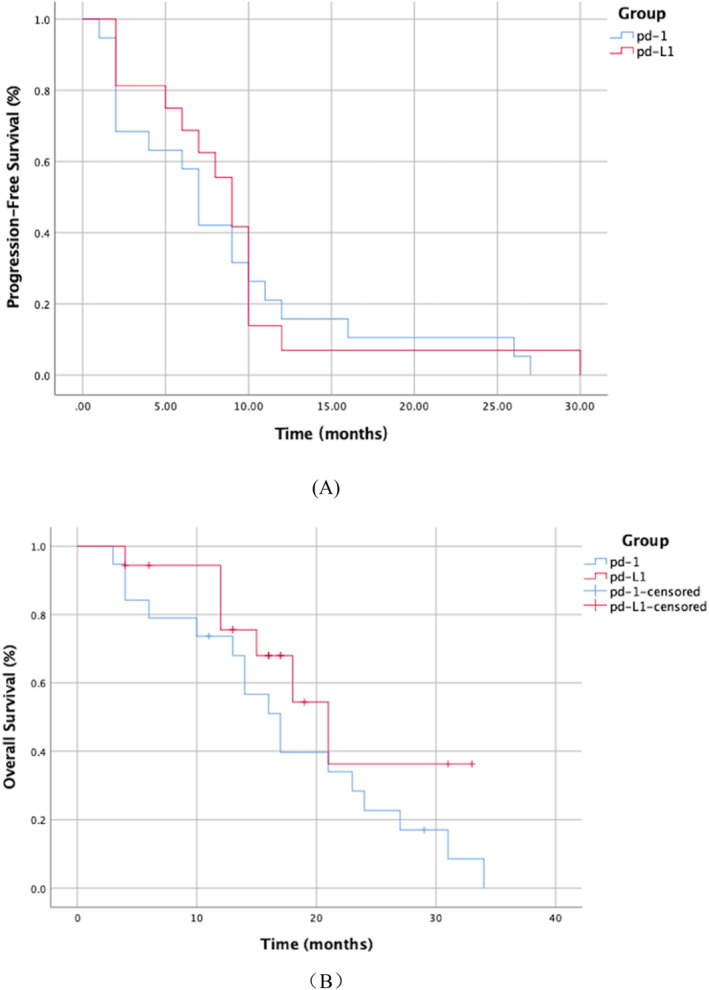
Survival Outcomes for Patients with Small‐Cell Lung Cancer: (A) Progression‐Free Survival (PFS). There was no significant difference in PFS observed between the PD‐1 and PD‐L1 treatment groups. (B) Overall survival (OS). Statistical comparison of OS between the PD‐1 and PD‐L1 groups revealed no significant differences.

The PFS spanned 8.5 months (95% CI, 5.1 to 11.9) for the PD‐1 group, slightly less than the 8.9 months (95% CI, 5.7 to 12.0) observed in the PD‐L1 group, though this difference was not statistically significant (χ^2^ = 0.205, *p* = 0.650). Similarly, the median OS recorded was 17.0 months (95% CI, 13.1 to 20.9) for the PD‐1 group compared to 21.0 months (95% CI, 14.3 to 27.7) for the PD‐L1 group, with no significant statistical discrepancy noted between the two (χ^2^ = 1.95, *p* = 0.163). (Table 4, Figure 1).

**TABLE 4:** Kaplan–Meier Survival Estimates for PFS and OS Among the 37 Enrolled Patients.

Efficacy		Group				χ^2^	P
		PD‐1(*n* = 19)		PD‐L1(*n* = 18)			
PFS						0.205	0.650
	Months (95% CI)	8.5	5.1–11.9	8.9	5.7–12.0		
	Median, months (95% CI)	7.0	5.6–8.4	9.0	7.1–10.9		
OS						1.95	0.163
	Months (95% CI)	17.5	12.9–21.9	21.8	16.1–27.6		
	Median, months (95% CI)	17.0	13.0–20.9	21.0	14.3–27.7		

*Note:* Survival analysis of the two groups revealed no statistically significant difference in OS and PFS between patients in the PD‐1 and PD‐L1 groups.

Abbreviations: CI: Confidence Interval; OS: Overall Survival; PFS: Progression‐Free Survival.

## 4 Discussion

SCLC is a notably aggressive and devastating malignancy. The introduction of immunotherapy represents a significant milestone in the clinical management of this disease, addressing challenges that have historically limited treatment success. This advancement has reshaped therapeutic strategies, offering new hope for improved patient outcomes in a landscape previously dominated by limited options [18, 19].

Phase III studies have demonstrated that integrating PD‐L1 inhibitors, such as atezolizumab and durvalumab, with chemotherapy significantly enhances OS in previously untreated ES SCLC patients, when compared to chemotherapy alone. In particular, the addition of atezolizumab to chemotherapy has extended OS to 12.3 months, compared to 10.3 months with chemotherapy alone (HR = 0.70; 95% CI, 0.54–0.91). Similarly, durvalumab when combined with chemotherapy extended OS to 13.0 months compared to 10.3 months for chemotherapy alone (HR = 0.73; 95% CI, 0.59–0.91). Reflecting these clinical outcomes, the FDA has sanctioned the use of atezolizumab and durvalumab, in conjunction with chemotherapy, as standard first‐line therapies for ES SCLC patients [20, 21]. In contrast, the role of PD‐1 inhibitors in improving OS and PFS for SCLC patients continues to be a subject of discussion. Notably, the Checkmate‐032 trial showed that nivolumab monotherapy markedly increased the ORR and the median DoR compared to controls. Consequently, the FDA has endorsed nivolumab as a monotherapy for metastatic SCLC patients who have not responded to platinum‐based chemotherapy and an additional treatment line, establishing it as a groundbreaking third‐line therapy for SCLC [22, 23]. Results from the phase II ECOG‐ACRIN EA5161 trial indicated that nivolumab combined with chemotherapy extended the median PFS to 5.5 months, in contrast to 4.7 months for chemotherapy alone, marking a statistically significant improvement. However, there was no statistically significant difference in OS observed between the two treatment modalities [24]. Furthermore, the KEYNOTE‐604 study, a randomized, double‐blind phase III trial, showed that while pembrolizumab in combination with chemotherapy improved PFS in patients, it did not significantly improve OS [25]. The body of research assessing the effectiveness of PD‐1/PD‐L1 inhibitors combined with chemotherapy in enhancing the prognosis of SCLC patients presents mixed results. In China, PD‐1 inhibitors are covered by medical insurance, making them a more cost‐effective and accessible option compared to the mostly imported PD‐L1 inhibitors. Consequently, PD‐1 inhibitors are the preferred choice for many SCLC patients in Gansu Province due to these advantages.

In our study, we assessed the efficacy and potential adverse reactions associated with the use of PD‐1/PD‐L1 inhibitors combined with chemotherapy in treating SCLC. The results demonstrated that patients treated with PD‐L1 inhibitors and chemotherapy experienced a median PFS of 8.9 months and a median OS of 21.0 months. Conversely, those treated with PD‐1 inhibitors and chemotherapy showed a median PFS of 8.5 months and a median OS of 17.0 months. No statistically significant differences were noted in either PFS or mOS between the two treatment groups. These findings support the potential of both PD‐1 and PD‐L1 inhibitors to improve survival outcomes in SCLC patients when used in conjunction with chemotherapy, aligning with observations from the CheckMate 032 trial [23].

However, it is imperative to emphasize that the increased incidence of adverse events remains a significant concern with combination therapies, especially those incorporating immune checkpoint inhibitors. SCLC patients receiving either treatment regimen exhibited varying degrees of adverse reactions, such as bone marrow suppression and gastrointestinal symptoms (e.g., diarrhea, vomiting). Notably, there were pronounced differences in the severity of these ADRs. For example, concerning bone marrow suppression, 47.4% (9 patients) of the PD‐1 group exhibited reactions of grade ≥ 3, whereas only 11.1% (2 patients) in the PD‐L1 group did. Regarding gastrointestinal reactions, all individuals in the PD‐L1 group experienced reactions with a severity of ≤ 2, while 31.6% (6 patients) in the PD‐1 group experienced grade ≥ 3 reactions. These findings align with those reported by Yu et al. [26]. Although both treatment approaches showed acceptable tolerability, our analysis suggests that SCLC patients treated with PD‐L1 inhibitors and chemotherapy may experience an enhanced quality of life.

## 5 Limitations

This retrospective single‐center study encompasses several inherent limitations: (1) The retrospective nature of the analysis might introduce selection and information biases, potentially influencing the outcomes. (2) The findings, derived from a single institution, may not be widely applicable to other settings, limiting the external validity of the results. (3) Given the relatively small cohort size, these findings necessitate further confirmation through larger, more diverse studies involving different ethnicities and geographic regions. Additionally, while Monika et al. [27] have noted an increased incidence of pneumonia associated with PD‐1 inhibitors in NSCLC patients as opposed to PD‐L1 inhibitors, such observations were not replicated in our analysis.

In conclusion, our findings demonstrate that integrating immunotherapy with chemotherapy is beneficial for SCLC patients, independent of whether PD‐1 or PD‐L1 inhibitors are used. While PD‐1 inhibitors tend to be associated with more severe adverse reactions compared to PD‐L1 inhibitors, they are generally well‐tolerated. Considering factors such as drug safety, efficacy, cost‐effectiveness, and patient preferences, PD‐1 inhibitors may be a viable alternative when PD‐L1 inhibitors are not suitable. Employing these treatment strategies could potentially improve survival rates and enhance the quality of life for SCLC patients.

## Author Contributions

All authors had full access to the data in the study and take responsibility for the integrity of the data and the accuracy of the data analysis. **Qichen Zhang:** conceptualization; formal analysis; data curation; methodology; writing – original draft preparation. **Xiaojuan Han:** investigation; data curation; formal analysis; validation. **Jiayi Liu:** investigation; validation; data curation. **Hui Qiao:** writing – review and editing (lead); supervision; funding acquisition.

## Ethics Statement

This retrospective analysis has been approved by the Ethics Committee of the First Clinical Hospital of Lanzhou University. During the design and implementation of the study, informed consent was obtained from participants wherever possible. Additionally, strict adherence to privacy protection principles was maintained in the analysis and reporting of data, with no identifiable personal information disclosed.

## Conflicts of Interest

The authors declare no conflicts of interest.

## Data Availability

The datasets generated during and/or analyzed during the current study are available from the corresponding author upon reasonable request.
